# Physicochemical, Quality and Flavor Characteristics of Starch Noodles with *Auricularia cornea* var. Li. Powder

**DOI:** 10.3390/foods13081185

**Published:** 2024-04-12

**Authors:** Yang Gao, Xinzhen Zhang, Ran Wang, Yue Sun, Xueling Li, Jin Liang

**Affiliations:** Key Laboratory of Jianghuai Agricultural Product Fine Processing and Resource Utilization of Ministry of Agriculture and Rural Affairs, Anhui Engineering Research Center for High Value Utilization of Characteristic Agricultural Products, College of Tea & Food Science and Technology, Anhui Agricultural University, Hefei 230036, China; gy17730218631@163.com (Y.G.); zxz3063739791@163.com (X.Z.); wr281423493@163.com (R.W.); yuesun@ahau.edu.cn (Y.S.); lxl72@126.com (X.L.)

**Keywords:** *Auricularia cornea* var. Li. (AU), starch paste, gel properties, structure, quality characteristics, starch noodles

## Abstract

*Auricularia cornea* var. Li., as an edible mushroom rich in various nutrients, could be widely used in noodle food. This study aimed to investigate the effect of *Auricularia cornea* var. Li. (AU) powder on the gel properties, structure and quality of starch noodles. Taking the sample without adding AU powder as a control, the addition of AU powder enhanced the peak viscosity, trough viscosity, final viscosity, breakdown, setback, peak time, gelatinization temperature, G′ (storage modulus) and G′′ (loss modulus). Meanwhile, the incorporation of AU powder significantly enhanced the stability of the starch gel structure and contributed to a more ordered microstructure also promoting the short-term aging of starch paste. In vitro digestion results displayed lower rapid digestibility (21.68%) but higher resistant starch content (26.58%) with the addition of AU powder and increased breaking rate, cooking loss, swelling index and a* and b* values. However, it decreased dry matter content and L*, particularly the reducing sugar content significantly increased to 4.01% (*p* < 0.05), and the total amino acid content rose to 349.91 mg/g. The GC-IMS library identified 51 VOCs, and the OPLS-DA model classified 18 VOCs (VIP > 1). Overall, the findings indicate that starch noodles with the addition of AU powder may provide greater nutritional quality, gel stability and starch antidigestibility.

## 1. Introduction

Starch noodles, as a staple food dominated by starch, are widely popular in Asia [1]. Starch noodles are different from other types of noodles (such as pasta and wheat noodles) because they are made from gluten-free starch (such as mung bean starch, pea starch and sweet potato starch). Moreover, starch noodles are convenient to eat, rich in taste and diverse in cooking methods [2]. Mung bean starch is considered the best raw material for preparing starch noodles, with excellent quality, smooth taste and resistance to cooking. However, due to its limited availability and high price, other starch sources are often used as partial or complete substitutes for starch noodles [3,4]. Sweet potato starch noodles are not only widely produced in China but also deeply influenced by consumers in East and Southeast Asia, playing an important role in global food production, such as being an effective substitute for expensive mung bean starch [5]. To address these issues, this study partially substituted starch by complexing commonly used mung bean starch and sweet potato starch in starch noodles.

*Auricularia cornea* var. Li. (AU), titled as *Yu Muer*, is a white mutant strain, homologous to food, with high yield and good quality [6,7]. Moreover, AU has excellent rehydration properties and is convenient for storing after drying [8]. AU is abundant in nutrients and has anti-inflammatory, anti-cancer, hypoglycemic and other functions [6,9,10]. Related research revealed that *A. cornea* var. Li. polysaccharides (APS) and enzymatically extracted APS both exhibited potential inhibitory effects on alcoholic liver disease [11]. A similar report explored the antidiabetic and antinephritic effects of AU in db/db diabetic models, and possible antioxidant and anti-inflammatory mechanisms, and detected the composition of AU [6]. Wang et al. investigated that *Auricularia cornea* var. Li. polysaccharides (ACP) not only could be used as a protein stabilizer and thickener in yogurt production but also enhanced the water-holding capacity, viscosity, firmness, and cohesiveness of yogurt, while imparting its characteristic mushroom flavor and promoting the activity of antioxidant enzymes [7]. However, using AU powder in food production to evaluate its effect on food quality, gel properties and flavor characteristics has been rarely reported to date.

The objective of this work was to utilize AU powder in starch noodles and to analyze and compare the pasting properties, rheological behavior, gel properties, microstructure, quality characteristics, amino acids and volatile organic compounds (VOCs) with and without the addition of AU powder, which might provide guidance for the potential utilization of AU powder in the food industry. 

## 2. Materials and Methods

### 2.1. Raw Material

**Figure 1:**
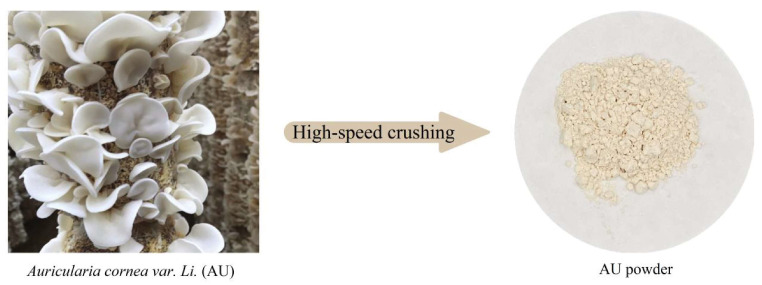
Flow chart of preparation of *Auricularia cornea* var. Li. (AU) powder.

*Auricularia cornea* var. Li. (AU) was supplied by Anhui Qin’er Fungi Co., Ltd. (Suzhou, China). The preparation for the material (dried AU) involved drying the fresh AU with an electric thermostatic blast drying oven (DHG-9053A, Shanghai Shenxian thermostatic equipment factory), where the drying parameters were as follows (temperature: 55 °C, time: 6 h). Subsequently, the material underwent high-speed crushing, and the resulting powder was sifted through 120 mesh sieves before being stored at room temperature for later use (Figure 1). Sweet potato starch (83% carbohydrate) was obtained from Sichuan Youjia Food Co., Ltd. (Chengdu, China), while mung bean starch (86.4% carbohydrate) was acquired from Shandong Jincheng Co., Ltd. (Zhaoyuan, China). The sodium carboxymethyl cellulose (food-grade) originated from Zhejiang Yinuo Biotechnology Co., Ltd. (Lanxi, China), and the sodium alginate (food-grade) was obtained from Henan Qiaoshou Food Additives Co., Ltd. (Zhengzhou, China). Salt (food-grade) was supplied by China Salt Dongxing Salt Co., Ltd. (Chuzhou, China). α-amylase (CAS: 9001-19-8, 100 U/mg) and gluco-amylase (CAS: 9032-08-0, 100 U/mg) were purchased from Yuanye Biotechnology Co., Ltd. (Shanghai, China). All other reagents were analytical grade. 

### 2.2. Preparation of Starch Slurry and Starch Noodles

#### 2.2.1. Preparation of Starch Slurry

Starch slurry was prepared following the method, with some modifications, described in [12]. The detailed process for preparing the starch slurry was as follows: a starch base was created using a 1:2 ratio of sweet potato starch to mung bean starch. Other additions were proportionally adjusted based on this starch base. Sodium carboxymethyl cellulose was added at 0.6%, sodium alginate at 0.4%, table salt at 0.6% and AU powder at 4.8%. The detailed acquisition of additions is summarized in the Supplementary Materials (Tables S1 and S2). These raw materials were fully mixed to achieve a uniform premixed material. Subsequently, water was added to the premixed material in a ratio of 13:6 (water to starch), creating a well-mixed and properly dissolved mixture. This slurry was then fully blended using a mixer for use in the experimental group. The control group was prepared without the addition of AU powder. 

#### 2.2.2. Preparation of Starch Paste

First, place the uniformly mixed starch slurry (20 g) and five glass beads into a 50 mL conical flask and then place them into a water bath thermostatic oscillator for gelatinization. Repeat three times for each sample. The measurement parameters were as follows: the vibration was set to reciprocating with a rotation rate of 250 r/min and heated at 95 °C for 20 min.

#### 2.2.3. Preparation of Starch Noodles

Starch noodles were prepared on the basis of [12]. The starch slurry was spread in a rectangular stainless steel tray and steamed in a steam boiler for 180 s to form a starch sheet. After steaming, the starch sheet was allowed to cool naturally. Subsequently, the sheet was taken out and cut into strips, creating starch noodles. These starch noodles were dried twice in an oven before being used as the experimental group. The control group was prepared without the addition of AU powder.

### 2.3. Determination of Nutritional Components

Essential nutrients were determined for all samples according to national standards for food safety, which included total starch, protein, ash, fats, soluble dietary fiber, insoluble dietary fiber and reducing sugars. Total starch was determined through acid hydrolysis methods, proteins were measured by the Kjeldahl method, ash by the incineration method, fats utilized the Soxhlet extraction method, while soluble dietary fiber and insoluble dietary fiber were determined by enzyme gravimetric method and reducing sugars by Fehling reagent (AOAC) [13]. 

### 2.4. Gel Properties

#### 2.4.1. Pasting Properties

Accurately weigh the sample (3.0 g) and place it into the special aluminum box of the rapid viscosity analyzer (RVA 4800, Perten, Newport Co. Ltd., Killara, Australia). Then distilled water (25.0 g) was added, fully dispersed with a rotary slurry and measured. The standard 1 program involved the following steps: the initial temperature was 50 °C, the samples were kept at 50 °C over 120 s, the temperature was heated to 95 °C at 6 °C/min, the samples were kept at 50 °C over 300 s, and then the temperature was decreased to 50 °C at 6 °C/min and the samples were kept at 50 °C over 120 s. At first, the stirring speed was 960 r/min for 10 s, and then was kept at 160 r/min [14,15]. 

#### 2.4.2. Rheological Properties

The samples were derived from starch paste obtained by RVA gelatinization, then analyzed for rheological properties by a rheometer fitted with Peltier elements (DHR-1, TA Instruments, New Castle, DE, USA). The dynamic and static shear modeling was performed with a parallel plate geometry system (diameter: 40 mm, gap: 1 mm), where the test temperature was set to 25 °C. An appropriate amount of starch paste (2.0–3.0 g) was then placed on the Peltier plate of the rheometer and balanced for 3 min prior to the rheological test [16,17]. During the test, the starch paste was positioned between the parallel plates. The edges of the starch paste were carefully trimmed and methyl silicone oil was used to seal the edges to prevent water loss during the trial.

##### Dynamic Frequency Sweep

The linear viscoelasticity of the sample was evaluated by varying amplitudes, employing the following test parameters: a fixed frequency of 1 Hz, a strain range from 0.01% to 100% and an ambient temperature of 25 °C. Through analysis of the results, a strain value of 2% was determined, affirming that the sample was within its linear viscoelastic range [17]. 

The sample underwent shear at a 2% strain during a frequency sweep ranging from 0.1 to 10 Hz, all at an ambient temperature of 25 °C. This test provided data for the storage modulus (G′), loss modulus (G′) and loss tangent (Tan δ = G′′/G′).

##### Static Rheological

The apparent shear viscosity of the gelatinized starch slurry was tested at an ambient temperature of 25 °C within a range of shear rates from 0.1 to 10 1/s. The outcomes expressed that the apparent viscosity varied with the shear rate under stable conditions [18].

#### 2.4.3. Gel Textural Properties

The method for determining the gel texture was adapted from the literature [19]. Following the RVA determination, the pasted samples were collected in 30 mL measuring cups and refrigerated at 4 °C for 24 h. Subsequently, the gel samples were brought to room temperature and subjected to texture analysis using a Texture Analyzer (Stable Micro Systems, Goldalming, UK) equipped with a P/0.5 probe. The pre-test, test, and post-test speeds were set to 1 mm/s each. A trigger force of 5 g and strain of 30% were applied.

#### 2.4.4. Freeze–Thaw Cycle (FTC) and Syneresis Measurement 

The determination of syneresis was conducted with certain modifications based on the method in [19,20]. The gel samples obtained after gelatinization using RVA were collected in centrifuge tubes. The tube was then placed in a refrigerator at −20 °C for 24 h. Afterward, it was thawed for half an hour in a water bath at 30 °C. Subsequently, the tube was centrifuged at 2250× *g* for 15 min. The apparent aqueous layer was discarded. This process constituted one freeze–thaw cycle (FTC-1). The procedure was replicated for 7 freeze–thaw cycles. The syneresis was calculated as in Equation (1):

(1) $$Syneresis\;(\%)=\frac{Weight\;of\;liquid\;separated\;from\;gel\;(g)}{Total\;weight\;of\;gel\;before\;centrifugation\;(g)}\times 100$$

### 2.5. Structural Properties of Starch Noodles

#### 2.5.1. Low-Field NMR (LF-NMR) Measurement

The 18 MHz LF-NMR analyzer (NMI20-15, Shanghai, China) was applied to determine the transverse relaxation time of water protons in starch noodles [21,22]. The starch noodle samples were placed into NMR tubes. Following the Q-FID calibration program, the CPMG (Carr-Purcell-Meiboom-Gill) pulse sequence was employed as the testing sequence. The CPMG pulse sequence was set to 8 scans, an echo number of 8000 and an echo time of 0.5 ms. The image was inverted using the device software.

#### 2.5.2. Scanning Electron Microscopy (SEM) 

Scanning electron microscopy (SEM, JSM-6490LV, JFOL Ltd., Tokyo, Japan) was used to scan the surface and cross-section of starch noodles, with the test conducted at an accelerating voltage of 5.0 kV and the magnifications of 100×, 500× and 4000×, respectively. Microscopic images at various magnifications were presented in the figures. Samples with suitable magnification were selected as representative micrographs [23].

#### 2.5.3. Fourier Transform Infrared Spectroscopy (FTIR) 

In an agate mortar, the powder obtained from starch noodles (powder sieved by 200 meshes) was mixed with KBr (100 mg of powder per 150 mg of KBr) and ground evenly. The mixture was then compressed into thin tablets using a tablet press. The IR spectra were scanned in the range of 4000–500 cm^−1^ and the resolution was 4 cm^−1^. The IR spectra of starch noodle samples were corrected and experimental curves were generated using Origin 9.11 software [23].

#### 2.5.4. In Vitro Digestibility 

The determination of in vitro digestion characteristics was based on the references [22,24]. Initially, the starch noodles were crushed into sample powder, and then 0.2 g of the sample powder was weighed and placed into a centrifuge tube. A solution of sodium acetate buffer (5 mL, pH 5.2) was boiled for 10 min and added to the centrifuge tube. After cooling in a 37 °C water bath, a mixture of 16 mL of a-amylase and 4 mL of glucoamylase was prepared and added to the centrifuge tube. The enzyme solution was incubated at 37 °C. Subsequently, 1 mL of the sample solution at different time points (0, 10, 20, 30, 60, 90, 120 and 180 min). To stop enzymatic digestion, 4 mL of 95% ethanol was quickly added to the collected samples, followed by centrifugation at 2250× *g* for 5 min. The supernatant was collected, and the DNS method was used to determine the glucose contents [25].

The hydrolysis rate and the content of rapidly digestible starch (RDS), slowly digested starch (SDS) and resistant starch (RS) were calculated using the formula below [22,24,25].

(2) $$RDS\left(\%\right)=\frac{\left[\left(T_{20}-FG\right)\times 0.9\right]}{TSC}\times 100$$

(3) $$SDS\left(\%\right)=\frac{\left[\left(T_{120}-T_{20}\right)\right]}{TSC}\times 100$$

(4) $$RS\left(\%\right)=\frac{\left[TSC-\left(RDS+SDS\right)\right]}{TSC}\times 100$$

where FG means the glucose content in starch before digestion (mg), TSC means the total starch content of the sample (mg), T_20_ means the Glucose content generated at 20 min of digestion (mg) and T_120_ means the Glucose content generated at 120 min of digestion (mg).

The starch digestion profile usually conforms to the first-order kinetic equation, as follows [22]:

(5) $$C_{t}=C_{\infty }\left(1-e^{-kt}\right)$$

where C_t_ represents the hydrolysis rate of starch when the digestion time is t, C_∞_ represents the equilibrium concentration of hydrolyzed starch at infinite time and k is the kinetic constant of starch hydrolysis, and t represents the digesting duration. 

### 2.6. Quality Characteristics and Volatile Compounds (VOCs) of Starch Noodles

#### 2.6.1. Cooking Properties

The breaking rate (BR), cooking loss (CL), swelling index (SI) and dry matter content (DMC) of the samples were determined based on the method of Yang et al. with modifications [1,23]. The samples were approximately 10 cm long and dried in an oven at 105 °C for 28 h. Subsequently, the sample was cooked in 800 mL of distilled water for 12 min. After cooking, the samples were rapidly cooled and the water adhering to the surface of the starch noodles was absorbed using blotting paper. The cooked samples were then placed in an oven at 105 °C and dried to a constant weight. The calculated equations were as follows:

(6) $$Breaking\;rate\;\left(\%\right)=\frac{Strands\;of\;broken\;noodles}{Strands\;of\;uncooked\;broken\;noodles}\times 100$$

(7) $$Cooking\;loss\left(\%\right)=\frac{\left(M_{1}-M_{3}\right)}{M_{1}}\times 100$$

(8) $$Swelling\;index\left(\%\right)=\frac{M_{2}}{M_{3}}\times 100$$

(9) $$Dry\;matter\;content=\frac{M_{1}}{M_{0}}\times 100$$

where M_0_ = weight of sample (5.0 g starch noodles), M_1_ = mass of starch noodles after 28 h of drying (g), M_2_ = mass of starch noodles after cooking (g) and M_3_ = weight of starch noodles dried to constant weight (g).

#### 2.6.2. Color Measurement

All the starch noodles were cut into uniform strips, which were then neatly arranged. The color of the samples was determined using a colorimeter (CR-400, Konica Minolta, Tokyo, Japan). The Hunter colorimetry reflectance values L* (white/black), a* (red/green) and b* (yellow/blue) were used to measure the apparent color of the starch noodles [26].

#### 2.6.3. Determination and Scoring of Hydrolyzed Amino Acids

The determination of hydrolyzed amino acids was modified following the method of Li et al. [27,28], where 100 mg samples (dry powder) were placed into the hydrolysis tube, then 10 mL of 6 mol/L HCL (analytically pure AR) was added and sealed by filling with N_2_. The hydrolysis tube was placed in an oven at 105 °C for 24 h, then, the hydrolyzed samples were diluted to 50 mL with ultrapure water (Watson’s) and 1 mL of the samples (solution) was taken into a small beaker and placed in a vacuum drying oven at 60 °C until dry. Then, 1 mL of 0.02 mol/L HCL (superior grade GR) was added to the beaker, 1 mL of the sample (solution) was taken in a small beaker and dried in a vacuum drying oven at 60 °C and 1 mL of 0.02 mol/L HCL (superior grade GR) was added to the beaker to re-dissolve the solution, which was then passed through a disposable aqueous membrane of 0.22 μm. Finally, the amino acid analysis was carried out by an automated amino acid analyzer (L-8900, Hitachi, Tokyo, Japan) for amino acid analysis and determination. The amino acid score was calculated based on the FAO/WHO recommendations [29].

#### 2.6.4. Determination of VOCs by GC-IMS

The GC-IMS analytical method was slightly modified according to the literature [30]. The volatile headspace components in the samples were determined using GC-IMS (FlavourSpec, G.A.S, Dortmund, Germany) for the analysis of volatile organic compounds (VOCs). The specific analysis parameters were set as follows: the sample powder (2.0 g) was transferred into the headspace vial (20 mL). For automatic headspace injection, the injection volume was 200 μL. The incubation time was 15 min, with an incubation temperature of 80 °C, an injection needle temperature of 85 °C and an incubation speed of 500 rpm. 

The GC-IMS parameters included a chromatographic column (MXT-WAX, 30 m × 0.53 mm i.d., 1.0 μm df, RESTEK, Newport Co. Ltd., Australia) with a column temperature of 60 °C. N_2_ was used as the carrier gas, and the IMS detector temperature was set to 45 °C. Under the gas chromatography conditions, the carrier gas (N_2_) was maintained at a constant flow rate of 150 mL/min. The initial carrier gas flow rate was 2 mL/min for 2 min, followed by a linear increase to 10 mL/min over 5 min and maintenance for 20 min. Finally, the flow rate was linearly increased to 100 mL/min over 25 min and maintained for 5 min.

### 2.7. Statistical Analysis 

Data analysis was conducted using IBM SPSS 25.0 software (SPSS, Chicago, IL, USA) for statistical purposes. The samples were analyzed from different perspectives using the FlavourSpec matched analysis software (VOCal, Reporter, Gallery Plot, Dynamic PCA) in GC-IMS. VOCal (spectral analysis, as well as qualitative and quantitative measurement of data), Reporter (direct comparison of spectral differences), Gallery Plot (visual and quantitative comparison of VOCs variations) and Dynamic PCA (for sample clustering analysis and rapid classification of unknown sample categories). SIMCA-P 14.1 software (Umetrics, Umea, Sweden) was used to perform orthogonal PLS-DA (OPLS-DA) to identify VOCs with variable importance (VIP) value > 1.0. Analysis of variance (ANOVA) was employed, followed by post hoc multiple comparisons using the LSD and Duncan tests. The options for description and homogeneity of variance test were utilized as well. All results are presented as the mean ± standard deviation, with all tests repeated in triplicate. Experimental curves were generated using Origin 2021 software. A significance level of *p* < 0.05 was considered statistically significant.

## 3. Results and Discussion

### 3.1. Nutritional Composition Analysis

As shown in Table 1, the contents of total starch, protein, ash, fat, soluble dietary fiber, insoluble dietary fiber and reducing sugar in the control group were 84.09%, 0.94%, 0.71%, 0.15%, 0.52%, 5.05% and 0.27%, respectively. However, the total starch content of the experimental group decreased significantly. The contents of ash, soluble dietary fiber, insoluble dietary fiber and reducing sugar increased significantly. In particular, the content of the reducing sugar increased to 4.01%, which agreed with the reported findings [6]. This indicated that the addition of AU powder not only increased the content of the reducing sugar in starch noodles but also improved the nutritive value; it enhanced the nutritional quality of starch noodles.

### 3.2. Pasting Properties

**Figure 2:**
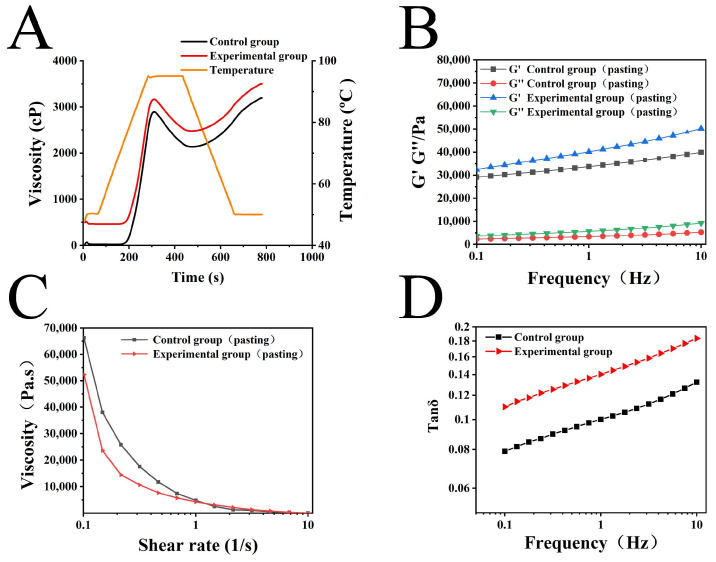
Pasting and rheological properties of the samples. (**A**) Pasting curves of samples; (**B**) Frequency sweeps of starch paste from 0.1 to 10 Hz. G′: storage modulus, G′′: loss modulus; (**C**) Steady shear flow curves of starch paste; (**D**) The relationship between tanδ and frequency of starch paste.

In the studies of starch pasting properties, viscosity change is an essential indicator for assessing the behavior of starch in different applications. As presented in Figure 2A and Table 2, the viscosity of starch increased with the addition of AU powder, and the trough viscosity (TV) increased most significantly. These results revealed that the addition of AU powder significantly affected the physical properties of starch, particularly by promoting the swelling of starch granules and the release of amylose, which modified the pasting properties of starch. The addition of AU powder facilitated the water absorption and swelling of the starch granules, leading to the release of amylose from the granules into the solution, subsequently increasing the concentration of amylose in the solution. During the cooling process, amylose molecules formed a tighter network structure through enhanced intermolecular interactions (such as hydrogen bonding), which facilitated the formation of gels, thereby further increasing the TV. Similar studies have also indicated that the addition of Mesona chinensis polysaccharides can improve the gelatinization temperature (GT) of starch [14]. Zhou’s findings revealed that there was no significant difference (*p* < 0.05) in the peak viscosity (PV) of different concentrations of oligosaccharides, whereas it was in accordance with the results of this study on the PV values [31].

The pasting parameters are presented in Table 2. The increase in the breakdown (BD) value reflects the higher sensitivity of starch paste with AU powder added to the shearing force, which means lower stability during processing. Ma et al. revealed that the BD was correlated with the PV, where increased PV values demonstrated greater granule swelling and severity of rupture, as well as severe amylose leaching [14,16]. In this study, when AU powder was added, we observed not only the increase in PV values but also a significant increase in BD values. This further confirms that the addition of AU powder promotes the water absorption and swelling of starch granules. Moreover, this process was often accompanied by excessive swelling and rupture of the granules, thus leading to a greater release of amylose and amylopectin. The setback (SB) value represented the difference between final viscosity (FV) and TV. Meanwhile, the increase in SB value revealed the enhanced stability of the starch paste after cooling with the addition of AU powder. Higher SB values reflected the improved stability and recrystallization tendency of the starch paste during cooling and reheating, which was associated with the reinforced interactions between amylose molecules. To summarize, the addition of AU powder promoted the swelling of starch granules and the release of amylose, and improved the gel-forming ability of the starch paste, as reflected in the improvement in TV. Moreover, it also affected its processing performance, as illustrated by the increase in SB value. The findings have significant meaning for a better understanding of the effect of AU on the starch pasting properties, which provide valuable information for the development of starch materials and optimization for applications.

### 3.3. Rheological Properties

#### 3.3.1. Dynamic Rheological Properties

As displayed in Figure 2B, both the G′ (storage modulus) and G′′ (loss modulus) of different samples displayed a noteworthy increase as the frequency ranged between 0.1–10 Hz. Additionally, G′ consistently surpassed G′′, which indicated that the elastic modulus occupied the main position, and the samples were solid. While the subsequently prepared slurry was also in the solid gel state after gelatinization [32]. Comparatively, the experimental group exhibited higher G′ and G′′, indicating that the experimental group exhibited stronger hindrance to the flow of substances and showed a higher loss modulus in the dynamic rheology. This might be attributed to the addition of AU powder providing a certain amount of reducing sugars. The characteristic structure of reducing sugar -OH imparted greater hydrophilicity. Simultaneously, its molecular chain structure could entangle with amylose or amylopectin molecules, thereby bestowing the experimental group with higher viscoelasticity.

G′ and G′′ represented the elastic modulus and viscous modulus of the samples, respectively. The ratio between these two values was the loss angle (Tanδ), used to measure the viscoelasticity of the samples. As shown in Figure 2B, both the G′ and G′′ in the two samples increased. With the value of G′ remaining consistently greater than that of G′′. Additionally, Tanδ was less than 1 (Figure 2C). This pattern indicated a typical weak gel rheological mode, suggesting that the starch paste was a viscoelastic non-Newtonian fluid primarily exhibiting elastic properties [32]. The magnitude of tanδ could reflect the proportion of viscosity and elasticity in the gel system. A higher tanδ value indicated a larger proportion of viscosity within the system, suggesting significant fluidity in the gel. Conversely, a smaller tanδ value indicated greater elasticity, emphasizing more pronounced solid properties in the gel [33]. As observed in Figure 2C, the tanδ value was low, which indicated strong elasticity. Furthermore, in comparison to the control group, the experimental group exhibited higher viscosity but lower elasticity.

#### 3.3.2. Static Rheological Properties

As depicted in Figure 2D, the apparent viscosity of the samples decreased with increasing shear rate. This phenomenon was a characteristic example of shear thinning, indicating that all samples belonged to pseudoplastic fluid. However, the viscosity changes were significantly different due to the composition differences of the samples [34]. The control group exhibited a higher viscosity, which is probably attributed to its elevated content of amylose in the gel structure, resulting in increased shear stress and, consequently, higher viscosity. Conversely, the sample in the experimental group, due to the addition of AU powder, experienced a greater combination of reducing sugars and starch within the mixed system. This circumstance somewhat weakened its gel structure, leading to a reduction in apparent viscosity.

### 3.4. Gel Texture and Freeze–Thaw Stability Analysis

The essence of food texture pertained to the sensory manifestation of food structure and material properties. It was influenced by several factors, such as food composition, structure and state. To mitigate subjective biases, mechanical devices were commonly employed to subject samples to controlled deformations, simulating the mastication process in human teeth. This approach objectively reflected the textural attributes of the food. The effect of AU powder on starch gel texture properties at different time intervals is shown in Table 3. Among the critical parameters in texture analysis, hardness assumes particular significance as a fundamental indicator of sample quality. The hardness of the starch gel was primarily governed by the expansion force exerted by starch particles. The observed trend of an initial increase followed by a subsequent decrease in hardness over time highlights the complex interplay of various factors during the gelatinization process. Notably, the hardness of the starch gel was closely linked to the swelling of starch particles and the exudation of contents, which significantly influenced the overall gel matrix. During samples pasting, the addition of AU powder promoted the exudation of amylose from the starch particles. This molecular behavior, combined with the subsequent aggregation and rearrangement of starch molecules, fosters the formation of a robust gel network, facilitating the establishment of starch gels with higher hardness during the early storage period (1 day/3 days). Previous findings revealed that the addition of mulberry leaf polysaccharides retarded the degradation of starch after FTC in starch gels and stabilized the microstructure of starch gels [19]. As shown in Table 3, starch gels with added AU powder exhibited significant variation in cohesiveness during the initial period of storage (1 day/3 days). Such increased cohesiveness and the subsequent improved stability of the gel structure are most likely attributable to the AU polysaccharides contained in the added AU powders [35]. The hardness of the starch gels decreased significantly during the later storage stages (7 days/14 days). The decrease in hardness could be attributed to the prolonged aging of starch, resulting in a looser gel structure characterized by water exudation and reduced molecular interactions [19]. The comprehensive understanding of these intricate interactions between AU powder, starch gel structure and textural properties provided valuable insights for optimizing food formulations and processing techniques to enhance food quality and consumer satisfaction.

As illustrated in Table 4, the syneresis of the control group was 17.14% after one freeze–thaw cycle. It progressively increased with the number of freeze–thaw cycles, reaching its peak value after six cycles. Subsequently, the water precipitation rate decreased to 41.62%. This phenomenon suggested that freeze–thawing promoted synergism within the control group. However, this synergism tended to lead to a decrease in the quality of starchy foods during the freezing process [20]. With the increased freeze–thaw cycles, the syneresis of the experimental group gradually increased from 21.72% to 39.79%. After three freeze–thaw treatments, the water precipitation rate of the experimental group was lower compared to that of the control group. It indicated that the addition of AU powder effectively mitigated the phenomenon of dehydration and shrinkage, which improved the freeze–thaw stability of the gel. 

### 3.5. LF-NMR Analysis

**Figure 3:**
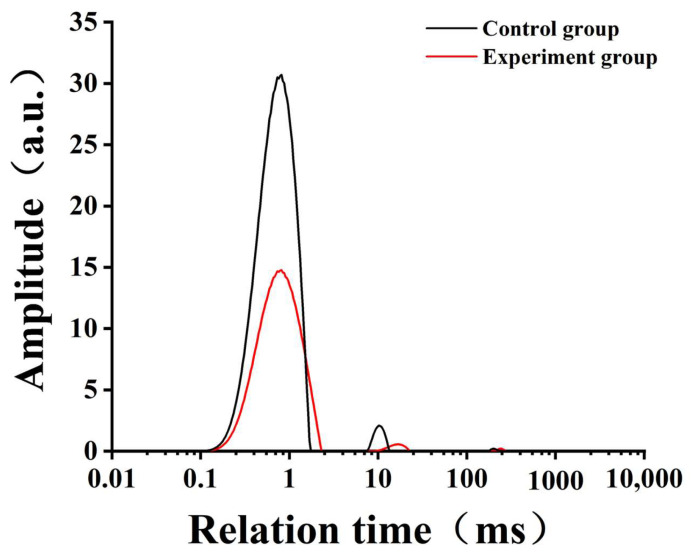
The transverse relaxation time (T_2_) of starch noodles.

LF-NMR, a rapid and efficient technique, was applied to investigate the distribution and migration of water molecules in food systems [36]. According to the water mobility, the water in the food matrix could be divided into three parts: T_21_ (bound water), T_22_ (semi-bound water) and T_23_ (free water) [22]. The morphology and distribution of water molecules were strongly associated with the structure and functional properties of gelatinized starch gels. The transverse relaxation time (T_2_) of starch noodles was exhibited in Figure 3: there was a big peak at 0.1–3 ms, a small peak at 6–30 ms and 150–300 ms, respectively. The transverse relaxation time presented the water freedom. Changes in the relaxation time distribution revealed the effect of AU powder on the distribution of various water groups within starch pastes, which meant the combined state and free movement degree of water under each state. The percentage of relaxation peak area of relaxation times T_21_, T_22_, T_23_ and P_21_, P_22_ and P_23_ are detailed in Table 5, which could be used to estimate various states of water [37]. As depicted in Figure 3, the starch paste contained the highest content of bound water, followed by semi-bound water and small free water. This indicated that the starch slurry formed a dense network structure after gelatinization and retrogradation, effectively impeding the outward migration of water. However, the addition of AU powder facilitated the migration of water; both T_21_ and T_22_ decreased, and the stability of the gel network structure decreased (Figure 3). The addition of AU powder promoted the swelling of starch, leading to the exudation of amylose and the interaction between amylose molecules. It promoted the short-term aging of starch and led to the reduction of bound water components to some extent.

### 3.6. SEM Test

**Figure 4:**
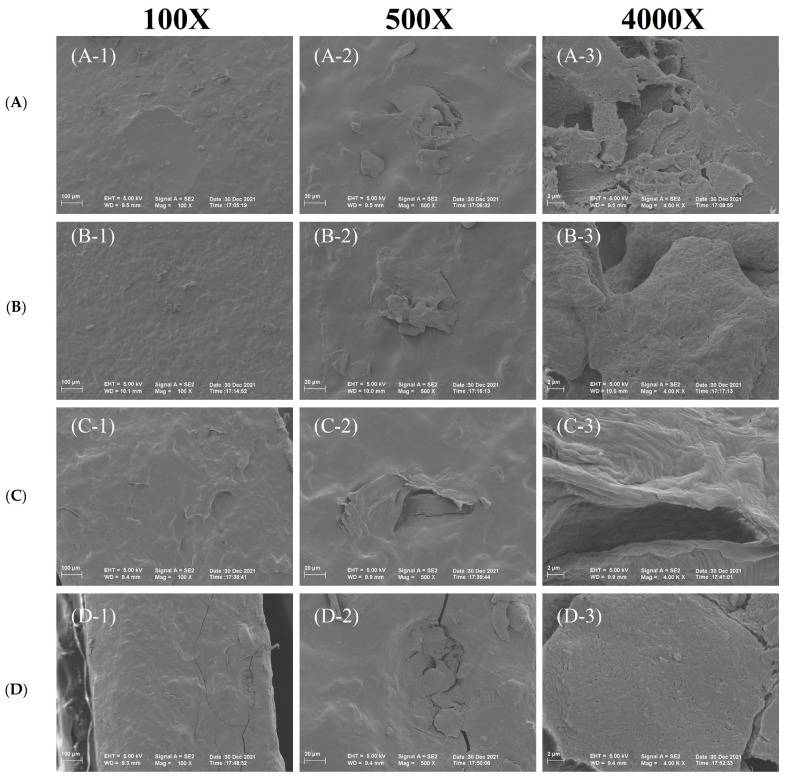
Micro-images of starch noodles. (**A**) The surface of the control group; (**B**) the surface of the experiment group; (**C**) the cross-section of the control group; (**D**) the cross-section of the experiment group.

In order to study the morphological features of starch noodles and distinguish differences between samples, the microscopic images of the samples were recorded by scanning electron microscopy [38]. The SEM images (Figure 4A–D) revealed the distinct differences between the samples. The experimental group exhibited smoother surfaces with numerous cracks on the cross-section, possibly attributed to glassy transitions and imbalanced shrinkage under the moisture-induced stresses [39]. Conversely, the control group displayed a characteristic bulk and lamellar ribbon structure in the cross-section [21,23]. The formation of this structure might be related to damage in the crystalline region and amylose exudation during the processing of starch slurry under hot and humid conditions. Additionally, new associations among starch chains occurred during cooling. Similar findings were reported by Yang et al. [23], showing the formation of small pores with a more uniform distribution. It indicated that the addition of AU powder greatly affected the cross-linked or phase separation behaviors. The addition of AU powder led to a denser and tightly structure, highlighting its role in promoting uniform water distribution and establishing a more orderly and stable microstructure [33]. According to the literature, when the addition of Lycium barbarum pulp (LBP) was 5%, the cross-sectional microstructure of noodles had the highest degree of connectedness. However, this finding is consistent with the findings of this paper (cross-sectional microstructure of starch noodles with added AU powder) [40].

### 3.7. FTIR Analysis 

**Figure 5:**
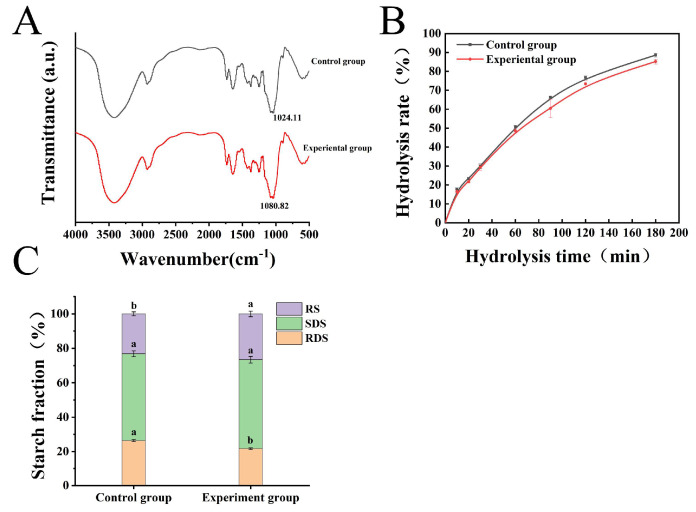
(**A**) FTIR spectra of starch noodles; (**B**) Hydrolysis rate of starch noodles; (**C**) The starch fraction distribution of starch noodles. Results are presented as means ± standard deviations (n = 3). Means with different letters in the columns are significantly different (*p* < 0.05).

The FTIR spectra of starch noodles are presented in (Figure 5A). The samples exhibited broad and strong absorbing peaks within the spectral range of 4000–500 cm^−1^. The change in the -OH stretching vibration represented the energy conversion among the O-H bonds, and the greater the wavenumber shift of the -OH peak, the weaker the intermolecular hydrogen bonding interactions [23].

In Figure 5A, 1047 cm^−1^ was strongly associated with the degree of order in the starch granules. It was extremely sensitive to changes in the crystal structures on the surface of the starch granules. Moreover, this portion of the amorphous area was more organized. However, 1022 cm^−1^ was strongly associated with the amorphous area in starch granules [15,41]. FTIR was sensitive to short-range order, and the ratios of 1047/1022 cm^−1^ could be used to characterize not only the change in short-range order of the starch structure but also related to the retrogradation degree of the starch in the sample [42]. The water molecules released from starch simultaneously as the double helices reorganize together during the aging process would contribute to the mobility of the starch chains. Moreover, the rearrangement of starch chains made the short-range ordering higher [43]. In this experiment, the addition of AU powder increased the absorbance and promoted the short-term aging of starch, which was consistent with the results in similar reports [14,42]. The IR_1047/1022_ values with and without the addition of AU powder were 1.01 and 1.02 (Table 6). The proportion of crystalline regions was higher in the experimental group and the formed double helix was more ordered. Meanwhile, the starch short-range order was higher, which promoted the short-term aging of the starch paste. The outcomes exhibited that the addition of AU powder promoted the short-term aging of starch paste, which corresponded to the outcomes of the RVA test.

### 3.8. In Vitro Digestibility

Figure 5B plots the percentages of digested starch versus the time of starch noodles within the first 180 min [15,22,24]. All samples displayed similar hydrolysis trends in their digestion curves. The addition of AU powder resulted in lower hydrolysis of the starch noodles [40]. Initially, the hydrolysis rate of the samples increased rapidly within 0–20 min and then increased relatively flatly from 20–120 min. Subsequently, the hydrolysis rate maintained lower rates after 120 min. Finally, at 180 min, the digestion rates with and without AU powder were 88.59% and 85.22%, respectively.

As depicted in Figure 5C and Table 7, the RDS and RS values of starch noodles also changed substantially with the addition of AU powder, with RDS decreasing from 26.46% to 21.68% and RS increasing from 23.14% to 26.58%. The result could be attributed to the following reasons: (1) The addition of AU powder promoted the dissolution of amylose and affected the mutual association of starch molecules; consequently, the addition of AU powder caused fluctuations in RDS content [22]; (2) AU contained more dietary fiber that was difficult to digest by digestive enzymes, which might contribute to the increase of RS content; (3) the addition of AU powder formed a denser microstructure which may possibly inhibit the movement of enzyme molecules to the granules and alter the swelling pattern of the starch granules during the pasting process, ultimately affecting the enzymatic digestion rate [44].

To better investigate the effect of AU powder addition on starch digestibility, the first-order kinetic equation was used to fit the starch hydrolysis rate curve, and the digestion kinetic parameters were calculated. Table 7 illustrated that the maximum degree of hydrolysis (C_∞_) and kinetic constant (k) obtained from starch digestion kinetics also had the same trend with the rate of starch hydrolysis. C_∞_ decreased significantly from 97.73% to 94.71%, and K decreased from 0.0128 min to 0.0123 min. As Hu et al. reported, with the addition of LBP, the starch hydrolysis rate of noodles decreased significantly (*p* < 0.05) [40].

### 3.9. Quality Characteristic 

#### 3.9.1. Cooking Properties

Cooking properties were used to evaluate the water absorption capacity and cooking resistance of starch noodles [9,23]. Cooking loss was used as an indicator to evaluate the cooking resistance of starch noodles. Meanwhile, the swelling index was used to assess the amount of water absorption capacity of starch noodles. As shown in Table 8, the experimental group exhibited a higher breaking rate and cooking loss of starch noodles, with values of 38.33% and 8.15%, respectively. The experimental group had a higher swelling index (514.18%), which could be attributed to the fact that the dietary fibers in the AU powder made the structure of the starch noodles itself more prone to water absorption. This facilitated the entry of water molecules from the outside into the starch noodles during the cooking process. Furthermore, the dry matter content of both types of starch noodles complied with the current national standard (GB/T 23587-2009 Vermicelli) [45]. Finally, although textural properties (taste, etc.) were not examined but they are important for consumer acceptance.

#### 3.9.2. Color Difference

As presented in Table 8, the L* (white) value of the experimental group was significantly reduced to 27.1 ± 1.07. It might be due to the fact that the addition of AU powder inhibited the swelling of the starch so that there were residual starch granules in the starch noodles. Moreover, the experimental group had higher a* and b* values. It could be attributed to the color properties of the added AU powder itself, affecting the variation of a* (red) and b* (yellow) of starch noodles.

#### 3.9.3. Hydrolyzed Amino Acid Analysis and Amino ACID Score

As shown in Table 9, the control group detected 13 amino acids, which included 5 essential amino acids and 8 non-essential amino acids. In contrast, 15 kinds of amino acids were detected in the experimental group, including 6 kinds of essential amino acids and 9 kinds of non-essential amino acids. In terms of amino acid content, the experimental group had the highest total amount of amino acids, totaling 349.91 mg/g. The content of 15 amino acids in the experimental group increased significantly compared with that in the control group.

**Figure 6:**
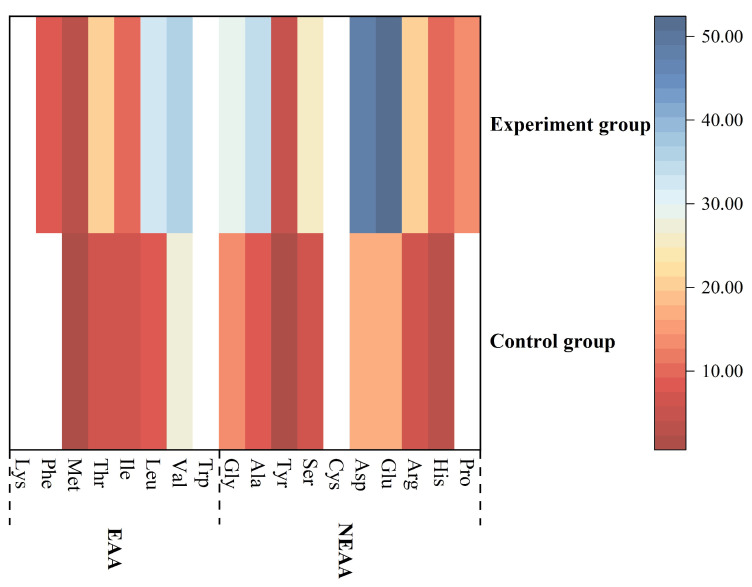
Essential amino acids (EAA) and non-essential amino acids (NEAA) contents of the samples.

This important aspect of evaluating the nutritional value of food was mainly measured by the content of essential amino acids. As illustrated in Figure 6, the experimental group had a higher content of five essential amino acids (Met, Thr, Ile, Leu, and Val). The findings revealed that the experimental group not only contained phenylalanine (Phe) but also promoted an increased in the total content of essential amino acids (EAA) in starch noodles. The Phe detected in this study was similar to the report of Wang et al. [6]. Furthermore, due to the inability of phenylketonuria (PKU) patients to metabolize phe, thus an intake of foods containing AU-associated needs to be strictly controlled for PKU patients [46,47].

In Figure 6, as compared to the experimental group, the control group had a lower content of the eight non-essential amino acids (Gly, Ala, Tyr, Ser, Asp, Glu, His and Arg). In addition, cysteine (Cys) was not detected in samples, but only proline (Pro) was detected in the experimental group. 

The figure presented that the addition of AU powder not only demonstrated the presence of proline (Pro) in AU but also promoted a higher total content of non-essential amino acids (NEAA) in starch noodles. The addition of AU powder promoted the increase of total essential amino acids and total non-essential amino acids in starch noodles, which indicated that AU possessed richer amino acids [6]. As shown in Table 10, it is calculated according to the reference amino acid composition of different consumer groups recommended by FAO/WHO [29]. As the outcomes indicated, for different age groups (pre-schoolchildren, schoolchildren and adults), the limiting amino acid was Val among the samples with or without the addition of AU powder. Compared with the non-adult group, Adult samples had higher estimates, particularly with the addition of AU powder. Nevertheless, this finding is consistent with the findings of Srimarut et al. [48]. Furthermore, we had higher estimated values with the added AU powder, particularly the Val values (2.81). 

#### 3.9.4. GC-IMS Detection

**Figure 7:**
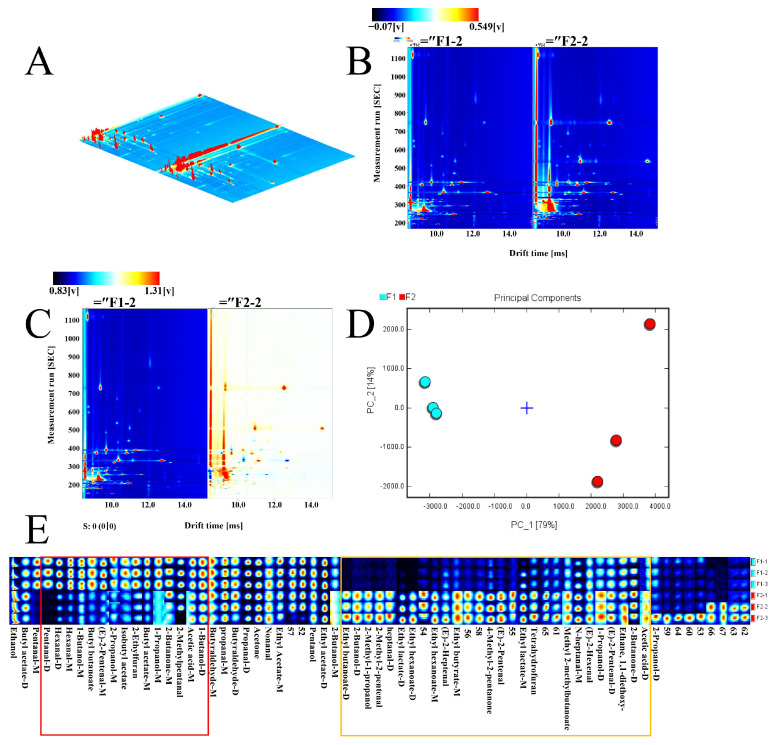
GC-IMS 3D spectrum of VOCs in the samples (**A**); GC-IMS spectra of volatile organic compounds in the samples (direct comparison) (**B**); GC-IMS spectra of volatile organic compounds in the samples (difference comparison) (**C**); principal component analysis of the samples (**D**); fingerprint gallery of VOCs in the samples (**E**).

F1 and F2 are used to represent the different samples, respectively (Figure 7B–E). Figure 7A presents a direct comparison of VOC differences between samples. However, for the sake of convenience in observation, the top view was utilized for the difference comparison plot (Figure 7B). In this plot, the entire spectrum depicted the VOCs of the sample. The normalized red vertical line on the left side of the spectrum represented the reactive ion peak (RIP). The vertical coordinate represented the retention time of gas chromatography (s), while the horizontal coordinate represented the ion mobility time. Each point on both sides of the RIP symbolized a VOC, with colors on the plot indicating substance concentration. Darker colors indicate higher concentrations, red color indicates high concentration and white color indicates low concentration. 

To compare the VOC distributions between samples, time regions of the main substances mentioned above were intercepted and exhibited in Figure 7C. The results revealed significant differences in VOCs among different samples. Specifically, the experimental group exhibited the most diverse VOC distributions, while the control group displayed comparatively less variation (Figure 7C). PCA analysis results indicated no overlap in volatile odor regions between samples. This shows that the VOCs of the samples differed significantly (Figure 7D). 

A visualization was made to investigate the differences in the composition of trace VOCs in samples (Figure 7E) [30]. The detailed peak volume data on volatile organic compounds are shown in the Supplementary Materials (Table S3). In this visualization, each row depicts all signal peaks selected from a single sample, while each column portrays signal peaks of the same VOC across different samples. By comparing the differences among the fingerprints (Figure 7E), the results demonstrate that the VOCs of samples with and without the addition of AU powder differ substantially. A total of 67 VOCs were identified (51 were specifically identified by the GC-IMS library, and 16 unidentified VOCs were assigned numerical designations). The composition aligned with the GC-IMS library, encompassing 18 aldehydes, 14 esters, 11 alcohols, 4 ketones, 2 acids, and 2 heterocyclics (Table 11) [49]. Compared with F1, the content of 28 VOCs increased (the area marked with a yellow rectangle), and the content of 16 VOCs decreased (the area marked with a red rectangle). As illustrated in the red box, 16 VOCs could be classified as aldehydes (pentanal-M, pentanal-D, hexanal-D, hexanal-M, E-2-pentenal-M, 2-methylpentanal), alcohols (1-butanol-M, 2-propanol-M, 1-propanol-M, 1-butanol-D), esters (butyl butanoate, isobutyl acetate, butyl acetate-M), acids (acetic acid-M), ketones (2-butanone-M), furans (2-ethylfuran). The yellow box was dominated by 28 VOCs could be divided into 7 aldehydes (2-methyl-2-pentenal, heptanal-D, E-2-heptenal, E-2-pentenal, N-heptanal-M, E-2-hexenal, E-2-pentenal-D), 3 alcohols (2-butanol-D, 1-propanol-D, 1-propanol-2-methyl), 7 esters (ethyl butanoate-D, ethyl lactate-D, ethyl hexanoate-D, ethyl hexanoate-M, ethyl butyrate-M, ethyl lactate-M, methyl 2-methylbutanoate), 1 acid (acetic acid-D), 3 ketones (4-Methyl-2-pentanone, Ethane-1-1-diethoxy-, 2-Butanone-D), 1 furans (tetrahydrofuran) and 6 unidentified VOCs. For further analysis, the following studies were, therefore, conducted.

#### 3.9.5. Distinction of Samples by OPLS-DA and Modeling Evaluation Analysis

**Figure 8:**
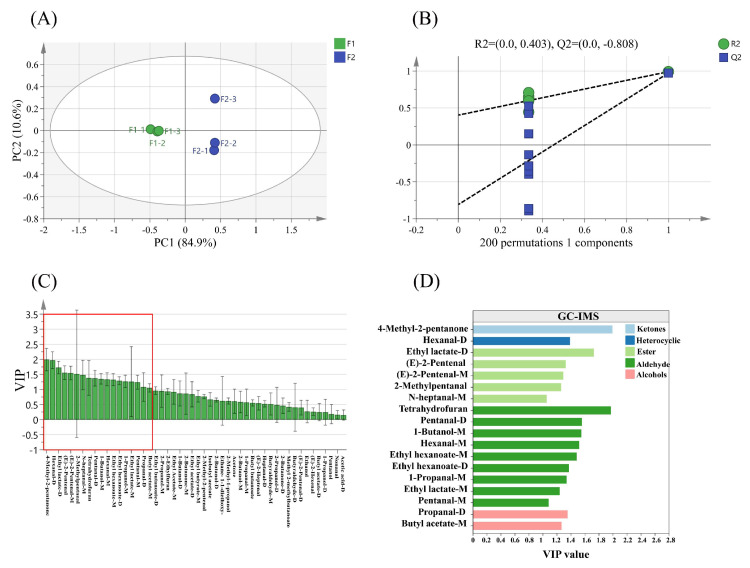
OPLS-DA analysis. Comparative analysis of VOCs between control and experimental samples based on OPLS-DA modeling. (**A**) OPLS-DA biplot (R2X = 0.955, R2Y = 0.992, Q2 = 0.976); (**B**) cross-validation by a 200-times permutation test (R2 = 0.403, Q2 = −0.808); (**C**) VIP-plot; (**D**) important VOCs (VIP > 1.0) among the samples determined by the GC-IMS. VOCs compounds marked in red (**C**) means VIP > 1.

OPLS-DA was a valid discriminant analysis statistical method for predicting the classification of samples. In order to better investigate the variation of volatile components in starch noodles, a further analysis was conducted with OPLS-DA. As presented in Figure 8A, the reliable OPLS-DA model revealed a significant difference between samples, demonstrating satisfactory variance and model predictive capability (R2X = 0.955, R2Y = 0.992, Q2 = 0.976). Additionally, the scatter plot of OPLS-DA scores effectively differentiated the samples and exhibited a classification effect similar to that of the PCA scatters. Moreover, the OPLS-DA model could further exclude irrelevant differences and achieve better separation of VOCs in the samples. The validation of model reliability was shown in Figure 8B. After 200 cross-validations (R2 = 0.403, Q2 = −0.808), all replacement test values for R2 and Q2 were lower than that of the original values. It indicated that the model was not overfitted and the constructed OPLS-DA model was stable and reliable. This analysis further supported the VOCs varied greatly between samples [50].

#### 3.9.6. Candidate Differential VOCs in Samples

After visualizing the 51 VOCs in the samples (Figure 7E), the contribution of each variable to the classification was quantified using the variable importance in the projection (VIP) approach. Based on the reliable OPLS-DA model construction (Figure 8A,B), the substances marked in red indicated that the VIP value of their VOCs was greater than 1 (Figure 8C) [33]. From the screening process, a total of 18 VOCs (VIP > 1) were identified, encompassing 9 aldehydes, 5 esters, 2 alcohols, 1 ketone and 1 heterocycle (Figure 8D). In this study, The control group identified nine key contributing VOCs (VIP > 1), including 6 aldehydes (hexanal-D, E-2-pentenal-M, 2-methylpentanal, pentanal-D, pentanal-M, hexanal-M), 2 alcohols (1-butanol-M, 1-propanol-M) and an ester (butyl acetate-M). Conversely, eight key contributing VOCs (VIP > 1) were identified by the experimental group, including 4 esters (ethyl lactate-D, ethyl lactate-M, ethyl hexanoate-M, ethyl hexanoate-D), 2 aldehydes (E-2-pentenal, N-heptanal-M), 1 ketone (4-methyl-2-pentanone) and 1 heterocycle (tetrahydrofuran). Notably, aldehyde (propanal-D) emerged as a common VOC (VIP > 1) in all samples.

### 3.10. Pearson Correlations Analysis

**Figure 9:**
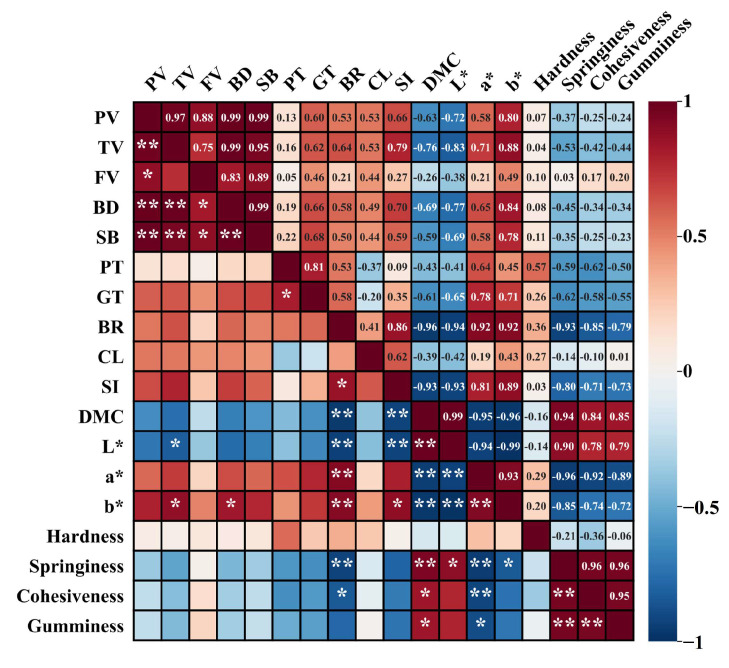
Pearson correlation coefficients between pasting parameters and starch noodles qualities. * and ** correlations represent the significant difference at *p* < 0.05 and *p* < 0.01 level, respectively. PV: peak viscosity; TV: trough viscosity; FV: final viscosity; BD: breakdown; SB: setback; PT: peak time; GT: gelatinization temperature; BR: breaking rate; CL: cooking loss; SI: swelling index; DMC: dry matter content.

The Pearson’s correlation analysis elucidated the correlation between pasting parameters and starch noodles qualities (Figure 9). The pasting parameters (PV, TV, BD and SB) were positively correlated with cooking and textural parameters (BR, CL, SI, a*, b* and hardness). However, the negative correlations with DMC, L*, springiness, cohesiveness and gumminess [51]. Meanwhile, FV positively correlated with BR, CL, SI, a*, b*, hardness, springiness, cohesiveness and gumminess, which was consistent with previous reports [51]. Furthermore, pasting parameters (PT, GT) were positively correlations with cooking and textural parameters (CL, DMC, L*, springiness, cohesiveness, gumminess), whereas negative correlated with BR, SI, a*, b* and hardness. 

The value of L* was considerably negatively related to the TV (r = −0.83*, *p* < 0.05), which agreed with previous reports [51]. Furthermore, The value of b* was significantly positively correlated with TV (r = 0.88*, *p* < 0.05) and BD (r = 0.84*, *p* < 0.05), respectively. These outcomes partially differed from reports of on modeling dried noodle quality [52]. In summary, the quality parameters (BR, CL, SI, a*, b*, hardness) of samples increased with the pasting parameters (PV, TV, BD, SB).

## 4. Conclusions

The effects of adding AU powder on the gel properties, structure and quality were investigated and the changes of VOCs in starch noodles were discussed by GC-IMS. The results show that the addition of AU powder contributed to the stability of starch gels. The structural characteristics indicate that the addition of AU powder was conducive to the formation of a more ordered microstructure, as well as a higher proportion of crystalline regions and a more ordered double helix structure. Notably, the addition of AU powder enriched the amino acid content of starch noodles. A total of 67 VOCs were detected by GC-IMS, and 51 VOCs were successfully identified. The OPLS-DA model classified 18 VOCs (VIP > 1), including 9 aldehydes, 5 esters, 2 alcohols, 1 ketone and 1 heterocycle. Pearson’s correlation analysis indicated that starch pasting parameters such as peak viscosity, trough viscosity, breakdown and setback should be the key indicators that synergistically affected the cooking characteristics of starch noodles. This study provided valuable information for the application of AU powder in starch noodles.

## Figures and Tables

**Table 1:** Nutritional composition of the samples.

Samples	Control Group	Experimental Group
Total starch (%)	84.09 ± 0.81 ^a^	68.87 ± 0.37 ^b^
Protein (%)	0.94 ± 0.004 ^a^	0.92 ± 0.02 ^a^
Ash (%)	0.71 ± 0.007 ^b^	0.78 ± 0.02 ^a^
Fats (%)	0.15 ± 0.004 ^a^	0.15 ± 0.003 ^a^
Soluble dietary fiber (%)	0.52 ± 0.03 ^b^	0.88 ± 0.03 ^a^
Insoluble dietary fiber (%)	5.05 ± 0.17 ^b^	6.89 ± 0.16 ^a^
Reducing sugar (%)	0.27 ± 0.002 ^b^	4.01 ± 0.06 ^a^

NOTE: Results are presented as means ± standard deviations (n = 3). Means within columns with different letters are significantly different (*p* < 0.05).

**Table 2:** Pasting parameters of the samples.

Samples	Control Group	Experimental Group
PV (cP)	2893 ± 50.51 ^a^	3093 ± 126.51 ^a^
TV (cP)	2138 ± 49.01 ^b^	2296 ± 60.01 ^a^
FV (cP)	3202 ± 83.76 ^a^	3489 ± 147.63 ^a^
BD (cP)	755 ± 1.53 ^b^	797 ± 66.94 ^a^
SB (cP)	1063 ± 34.93 ^a^	1193 ± 89.27 ^a^
PT (min)	5.14 ± 0.04 ^a^	5.20 ± 0.07 ^a^
GT (°C)	75.80 ± 0.00 ^a^	76.40 ± 0.52 ^a^

NOTE: Results are presented as means ± standard deviations (n = 3). Means with different letters in the columns are significantly different (*p* < 0.05). PV, TV, FV, BD, SB, PT and GT represent peak viscosity, trough viscosity, final viscosity, breakdown, setback, peak time and gelatinization temperature, respectively.

**Table 3:** Textural properties of the gel samples.

Samples	Hardness (g)	Springiness	Cohesiveness	Gumminess
CG-1d	223.39 ± 5.29 ^c^	0.55 ± 0.02 ^b^	0.72 ± 0.02 ^b^	151.70 ± 5.04 ^b^
CG-3d	308.77 ± 45.20 ^a^	0.72 ± 0.13 ^a^	0.39 ± 0.05 ^c^	121.56 ± 28.54 ^b^
CG-7d	288.00 ± 3.35 ^ab^	0.47 ± 0.02 ^b^	0.82 ± 0.08 ^ab^	235.98 ± 25.83 ^a^
CG-14d	249.34 ± 25.16 ^bc^	0.37 ± 0.01 ^c^	0.83 ± 0.02 ^a^	206.16 ± 18.18 ^a^
EG-1d	246.60 ± 3.83 ^b^	0.44 ± 0.02 ^b^	0.77 ± 0.01 ^b^	188.84 ± 2.52 ^b^
EG-3d	369.72 ± 71.69 ^a^	0.59 ± 0.02 ^a^	0.77 ± 0.03 ^b^	284.14 ± 50.18 ^a^
EG-7d	267.65 ± 12.32 ^b^	0.43 ± 0.03 ^b^	0.83 ± 0.01 ^a^	222.51 ± 13.81 ^b^
EG-14d	239.12 ± 11.90 ^b^	0.41 ± 0.01 ^c^	0.83 ± 0.04 ^a^	198.01 ± 18.61 ^b^

NOTE: Results are presented as means ± standard deviations (n = 3). Means with different letters in the columns are significantly different (*p* < 0.05). CG: control group, EG: experimental group.

**Table 4:** Effects of freeze–thawing cycles (FTC) treatment on freeze–thawing stability of the gel samples.

Syneresis (%)	Control Group	Experimental Group
FTC-1	17.14 ± 0.90 ^e^	21.72 ± 0.48 ^e^
FTC-2	28.14 ± 1.51 ^d^	29.00 ± 0.29 ^d^
FTC-3	36.40 ± 0.50 ^c^	32.79 ± 1.20 ^c^
FTC-4	39.60 ± 1.50 ^b^	36.32 ± 1.44 ^b^
FTC-5	42.13 ± 1.60 ^b^	38.33 ± 1.29 ^a^
FTC-6	45.71 ± 1.68 ^a^	38.65 ± 1.09 ^a^
FTC-7	41.62 ± 1.62 ^b^	39.79 ± 0.96 ^a^

NOTE: Results are presented as means ± standard deviations (n = 3). Means with different letters in the columns are significantly different (*p* < 0.05).

**Table 5:** Water distribution in the starch noodles.

Samples	Control Group	Experimental Group
T_21_ (ms)	0.93	0.81
P_21_ (%)	99.493	98.845
T_22_ (ms)	93.31	16.52
P_22_ (%)	0.484	1.057
T_23_ (ms)	982.84	443.22
P_23_ (%)	0.023	0.098

NOTE: P_21_, P_22_ and P_23_ represent the ratio of bound water, semi-bound water and free water to the total water, respectively. T_21_, T_22_ and T_23_ represent the relaxation time, respectively.

**Table 6:** The absorbance ratios of 1047/1022 cm^−1^ in starch noodles.

Samples	Control Group	Experimental Group
1047 cm^−1^	85.80	75.28
1022 cm^−1^	85.31	73.75
IR ratio 1047/1022 cm^−1^	1.01	1.02

**Table 7:** The digestibility fractions and digestive kinetics constants of the starch noodles.

Syneresis (%)	Control Group	Experimental Group
RDS (%)	26.46 ± 0.6 ^a^	21.68 ± 0.49 ^b^
SDS (%)	50.4 ± 1.69 ^a^	51.74 ± 1.9 ^a^
RS (%)	23.14 ± 1.18 ^b^	26.58 ± 1.64 ^a^
C_∞_ (%)	97.73 ± 0.51 ^a^	94.71 ± 1.03 ^b^
k (min^−1^)	0.0128 ± 0.0003 ^a^	0.0123 ± 0.0008 ^a^

NOTE: Results are presented as means ± standard deviations (n = 3). Means with different letters in the columns are significantly different (*p* < 0.05). RDS, SDS and RS indicate rapidly digestible starch, slowly digested starch and resistant starch, respectively. C_∞_: the equilibrium concentration of hydrolyzed starch at infinite time; k: the kinetic constant of starch hydrolysis.

**Table 8:** Cooking characteristics, dry matter content and color difference between the samples.

Samples	Control Group	Experimental Group
BR (%)	26.67 ± 2.89 ^b^	38.33 ± 2.89 ^a^
CL (%)	7.55 ± 0.47 ^a^	8.15 ± 1.01 ^a^
SI (%)	475.71 ± 6.84 ^b^	514.18 ± 15.14 ^a^
DMC (g/100g)	0.90 ± 0.003 ^a^	0.88 ± 0.001 ^b^
L*	37.27 ± 0.34 ^a^	27.1 ± 1.07 ^b^
a*	0.17 ± 0.02 ^b^	0.30 ± 0.04 ^a^
b*	0.22 ± 0.02 ^b^	1.05 ± 0.02 ^a^

NOTE: Results are presented as means ± standard deviations (n = 3). Means with different letters in the columns are significantly different (*p* < 0.05). BR: breaking rate; CL: cooking loss, %; SI: swelling index, %; DMC: dry matter content, g/100 g.

**Table 9:** Compositions of hydrolyzed amino acids in the samples.

Amino Acids (mg/g)	Control Group	Experimental Group
EAA		
Lysine (Lys)	ND	ND
Phenylalanine (Phe)	ND	8.89
Methionine (Met)	1.39	3.20
Threonine (Thr)	6.62	19.80
Isoleucine (Ile)	6.83	9.95
Leucine (Leu)	8.40	31.96
Valine (Val)	27.39	36.59
Tryptophan (Trp)	ND	ND
Total	50.62	110.39
NEAA		
Glycine (Gly)	13.37	29.33
Alanine (Ala)	8.30	34.82
Tyrosine (Tyr)	0.79	4.98
Serine (Ser)	6.29	25.00
Cysteine (Cys)	ND	ND
Aspartic acid (Asp)	16.52	48.47
Glutamic acid (Glu)	17.33	52.26
Arginine (Arg)	6.01	21.07
Histidine (His)	2.42	9.42
Proline (Pro)	ND	14.16
Total	71.03	239.53
TAA	121.66	349.91

NOTE: EAA: essential amino acids, NEAA: non-essential amino acids, TAA: total amino acids. ND: not detectable.

**Table 10:** The amino acid score estimated for the starch noodles with or without *Auricularia cornea* var. Li. (AU) powder.

Amino Acids	Reference ^a^ (mg/g Food Protein)	Control Group	Experimental Group
Pre-schoolchildren (2–5 years old)			
Histidine (His)	19	0.13	0.50
Isoleucine (Ile)	28	0.24	0.36
Leucine (Leu)	66	0.13	0.48
Lysine (Lys)	58	ND	ND
Methionine (Met) + cysteine (Cys)	25	0.06	0.13
Phenylalanine (Phe) + tyrosine (Tyr)	63	0.01	0.22
Threonine (Thr)	34	0.19	0.58
Valine (Val)	35	**0.78**	**1.05**
Schoolchildren (10–12 years old)			
Histidine (His)	19	0.13	0.50
Isoleucine (Ile)	28	0.24	0.36
Leucine (Leu)	44	0.19	0.73
Lysine (Lys)	44	ND	ND
Methionine (Met) + cysteine (Cys)	22	0.06	0.15
Phenylalanine (Phe) + tyrosine (Tyr)	22	0.04	0.63
Threonine (Thr)	28	0.24	0.71
Valine (Val)	25	**1.10**	**1.46**
Adults (>18 years old)			
Histidine (His)	16	0.15	0.59
Isoleucine (Ile)	13	0.53	0.77
Leucine (Leu)	19	0.44	1.68
Lysine (Lys)	16	ND	ND
Methionine (Met) + cysteine (Cys)	17	0.08	0.19
Phenylalanine (Phe) + tyrosine (Tyr)	19	0.04	0.73
Threonine (Thr)	9	0.74	2.20
Valine (Val)	13	**2.11**	**2.81**

NOTE: ^a^ FAO/WHO amino acid reference pattern [29] for three different age groups. Bold numbers denote the lowest amino acid score except for undetected. ND: not detectable.

**Table 11:** The information of identified volatile compounds by GC-IMS.

Category	No.	Compounds	GAS	Formula	MW	RI ^a^	Rt ^b^	Dt ^c^	Comment
Alcohols									
	1	1-Butanol	C71363	C_4_H_10_O	74.1	1153.3	422.751	1.1828	Monomer
	2	1-Butanol	C71363	C_4_H_10_O	74.1	1153.3	422.751	1.3871	Dimer
	3	Ethanol	C64175	C_2_H_6_O	46.1	945.4	271.931	1.135	
	4	Pentanol	C71410	C_5_H_12_O	88.1	1260.9	563.347	1.2594	
	5	1-Propanol	C71238	C_3_H_8_O	60.1	1046.6	330.38	1.1111	Monomer
	6	1-Propanol	C71238	C_3_H_8_O	60.1	1046.6	330.38	1.2547	Dimer
	7	2-Propanol	C67630	C_3_H_8_O	60.1	928.4	263.693	1.0924	Monomer
	8	2-Propanol	C67630	C_3_H_8_O	60.1	927.8	263.444	1.2188	Dimer
	9	2-Butanol	C78922	C_4_H_10_O	74.1	1047.1	330.749	1.1521	Monomer
	10	2-Butanol	C78922	C_4_H_10_O	74.1	1047.1	330.749	1.3182	Dimer
	11	2-Methyl-1-propanol	C78831	C_4_H_10_O	74.1	1046.5	330.348	1.3831	
Aldehyde									
	12	Hexanal	C66251	C_6_H_12_O	100.2	1098.3	367.534	1.2655	Monomer
	13	Hexanal	C66251	C_6_H_12_O	100.2	1099.7	368.822	1.5635	Dimer
	14	Pentanal	C110623	C_5_H_10_O	86.1	992.1	295.78	1.1939	Monomer
	15	Pentanal	C110623	C_5_H_10_O	86.1	991.3	295.333	1.421	Dimer
	16	2-Methylpentanal	C123159	C_6_H_12_O	100.2	767.8	197.452	1.2277	
	17	Propanal	C123386	C_3_H_6_O	58.1	833.5	222.289	1.0706	Monomer
	18	Propanal	C123386	C_3_H_6_O	58.1	832.4	221.851	1.1443	Dimer
	19	(E)-2-Pentenal	C1576870	C_5_H_8_O	84.1	1140.6	409.273	1.1189	Monomer
	20	(E)-2-Pentenal	C1576870	C_5_H_8_O	84.1	1143	411.809	1.3617	Dimer
	21	(E)-2-Heptenal	C18829555	C_7_H_12_O	112.2	1329.3	688.717	1.2568	
	22	(E)-2-Hexenal	C6728263	C_6_H_10_O	98.1	1227.1	513.589	1.1835	
	23	Ethane, 1,1-diethoxy-	C105577	C_6_H_14_O_2_	118.2	891	246.523	1.1283	
	24	Butyraldehyde	C123728	C_4_H_8_O	72.1	906.5	253.49	1.096	Monomer
	25	Butyraldehyde	C123728	C_4_H_8_O	72.1	911.3	255.73	1.2884	Dimer
	26	2-Methyl-2-pentenal	C623369	C_6_H_10_O	98.1	1143.6	412.414	1.5154	
	27	N-heptanal	C111717	C_7_H_14_O	114.2	1140.3	408.958	1.3215	Monomer
	28	Heptanal	C111717	C_7_H_14_O	114.2	1144.7	413.578	1.6802	Dimer
	29	(E)-2-Pentenal	C1576870	C_5_H_8_O	84.1	1099	368.147	1.366	
Esters									
	30	Ethyl lactate	C97643	C_5_H_10_O_3_	118.1	1355.4	747.429	1.1421	Monomer
	31	Ethyl lactate	C97643	C_5_H_10_O_3_	118.1	1355.4	747.429	1.5427	Dimer
	32	Ethyl hexanoate	C123660	C_8_H_16_O_2_	144.2	1243.7	537.384	1.3441	Monomer
	33	Ethyl hexanoate	C123660	C_8_H_16_O_2_	144.2	1242	534.913	1.8007	Dimer
	34	Butyl acetate	C123864	C_6_H_12_O_2_	116.2	1086.1	358.245	1.2371	Monomer
	35	Butyl acetate	C123864	C_6_H_12_O_2_	116.2	1086.1	358.245	1.6172	Dimer
	36	Ethyl Acetate	C141786	C_4_H_8_O_2_	88.1	893.8	247.767	1.0967	Monomer
	37	Ethyl Acetate	C141786	C_4_H_8_O_2_	88.1	900.4	250.753	1.3373	Dimer
	38	Isobutyl acetate	C110190	C_6_H_12_O_2_	116.2	1027.7	317.846	1.2417	
	39	Methyl 2-methylbutanoate	C868575	C_6_H_12_O_2_	116.2	1003	302.141	1.1832	
	40	Ethyl butyrate	C105544	C_6_H_12_O_2_	116.2	1046.5	330.348	1.1995	Monomer
	41	Ethyl butanoate	C105544	C_6_H_12_O_2_	116.2	1046.5	330.348	1.5592	Dimer
	42	Butyl butanoate	C109217	C_8_H_16_O_2_	144.2	1195.4	470.759	1.337	
	43	(E)-2-Pentenal	C1576870	C_5_H_8_O	84.1	1099	368.147	1.366	
Ketones									
	44	4-Methyl-2-pentanone	C108101	C_6_H_12_O	100.2	1022.1	314.223	1.1795	
	45	Acetone	C67641	C_3_H_6_O	58.1	850.8	229.302	1.115	
	46	2-Butanone	C78933	C_4_H_8_O	72.1	912.5	256.26	1.0602	Monomer
	47	2-Butanone	C78933	C_4_H_8_O	72.1	913	256.479	1.2453	Dimer
Acids									
	48	Acetic acid	C64197	C_2_H_4_O_2_	60.1	1483.1	1115.625	1.0557	Monomer
	49	Acetic acid	C64197	C_2_H_4_O_2_	60.1	1482.4	1113.194	1.1562	Dimer
Heterocycles									
	50	2-Ethylfuran	C3208160	C_6_H_8_O	96.1	990.4	294.886	1.2953	
	51	Tetrahydrofuran	C109999	C_4_H_8_O	72.1	869.9	237.316	1.0594	

^a^ Represents the retention index (RI) calculated using n-ketones C_4_–C_9_ as external standard on FS-SE-54-CB column. ^b^ Represents the retention time (RT) in the capillary GC column. ^c^ Represents the drift time (Dt) in the drift tube.

## Data Availability

The original contributions presented in the study are included in the article and Supplementary Material, further inquiries can be directed to the corresponding author.

## Supplementary Materials

The following supporting information can be downloaded at: https://www.mdpi.com/article/10.3390/foods13081185/s1, Table S1: Effect of *Auricularia cornea* var. Li. (AU) powder on comprehensive scores (texture score and sensory score); Table S2: Orthogonal experimental design of starch noodles quality improver; Table S3: Peak volume data for all compounds within each sample (Corresponds to Gallery Plot in GC-IMS).
