# Supplementary material for: Physicochemical, Quality and Flavor Characteristics of Starch Noodles with Auricularia cornea var. Li. Powder
Source: Foods. 2024 Apr 12;13(8):1185. doi: 10.3390/foods13081185 (PMC11048883; doi:10.3390/foods13081185)
Supplement: Supplementary file 1 [file foods-13-01185-s001.zip › foods-2899965-supplementary.pdf]

Table S1. Effect of *Auricularia cornea var. Li.* (AU) powder on comprehensive scores (texture score and sensory score).

Additive amount (%)	Comprehensive scores
1.6	62.62±3.90^{d}
3.2	65.09±4.07^{b}
4.8	71.41±4.28^{a}
6.4	63.31±4.39^{c}
8.0	56.26±4.58^{e}

[a] All values are means of triplicate determinations ± SD. Means within columns with different letters are significantly different ($p < 0.05$).

1.1.1 Texture determination

Firstly, starch noodles of 10 cm in length were cooked in boiling water. Following that, the noodles were transferred to a bowl of cold water for a duration of 20 seconds. Once removed from the water, absorbent paper was utilized to blot the surface moisture of the starch noodles. Subsequently, the starch noodles were spread out on the operating platform, ensuring its smoothness for the entire testing procedure.

To evaluate the texture of the starch noodles, a TA XTPlus Texture Analyzer (Stable Micro Systems, Goldalming, UK) equipped with a P/36R cylindrical probe was utilized. The pre-test, test, and post-test speeds were set at 2, 1, and 1 mm/s, respectively. A trigger force of 5 g and strain of 50% were applied.

1.1.2 Sensory evaluation

A sensory evaluation of the cooked starch noodles was carried out by a panel of 10 experts. The sensory evaluation criteria were based on a combination of GBT15682-2008 standards and were further adjusted to align with the specific

characteristics of the starch noodles. This evaluation encompassed aspects such as apparent color (20 points), visual appearance (20 points), aroma (20 points), and taste (40 points), with a maximum cumulative score of 100.

As displayed in Table S1,using comprehensive scores (texture score and sensory score) as scoring indicators, the optimal addition amount of *Auricularia cornea var. Li.* (AU) powder was determined to be 4.8%.

Table S2. Orthogonal experimental design of starch noodles quality improver.

Batch	Factor			Response
	CMC-Na	SA	TS	
1	1	1	1	0.358
2	1	2	2	0.367
3	1	3	3	0.308
4	2	2	3	0.412
5	2	3	1	0.391
6	2	1	2	0.382
7	3	3	2	0.302
8	3	1	3	0.283
9	3	2	1	0.322

[a] CMC-Na: Sodium carboxymethyl cellulose, SA: sodium alginate, TS: Table Salt.

[b] The response value represents the difference between cohesiveness and resilience.

1.2 Orthogonal optimization design

Building upon the results of the single-factor test, three key factors were chosen for further optimization testing: CMC-Na addition (%), SA addition (%), and TS addition (%). Simultaneously, three distinct levels were established for each of these factors, also based on the single-factor approach. Referring to Table S2, the optimal addition amounts of three improvers were obtained as follows: CMC-Na (0.6%), SA (0.4%), and TS (0.6%).

Table S3. Peak volume data for all compounds within each sample (Corresponds to Gallery Plot in GC-IMS).

Compounds	Category	F1-1	F1-2	F1-3	F2-1	F2-2	F2-3
acetic acid-M	Acids	605	576	552	502	489	502
Ethyl lactate-M	Esters	167	626	734	985	981	976
Ethyl lactate-D	Esters	16	119	157	725	883	974
Ethyl hexanoate-M	Esters	89	112	119	563	544	579
Ethyl hexanoate-D	Esters	13	14	15	405	413	512
1-Butanol-M	Alcohols	860	813	801	410	416	370
1-Butanol-D	Alcohols	974	927	908	781	764	822
Hexanal-M	Aldehyde	756	726	707	312	314	298
Hexanal-D	Aldehyde	1755	1782	1786	916	861	800
Ethanol	Alcohols	1724	1740	1737	1814	1780	1771
butyl acetate-M	Esters	537	546	547	294	295	261
butyl acetate-D	Esters	182	194	206	230	207	167
4-Methyl-2-pentanone	Ketones	539	559	538	1576	1583	1626
Pentanal-M	Aldehyde	418	402	396	33	31	34
pentanal-D	Aldehyde	478	491	470	24	19	14
2-Ethylfuran	Heterocycles	536	556	540	368	345	323
2-Methylpentanal	Aldehyde	1117	1401	1363	1136	1164	50
propanal-M	Aldehyde	935	964	964	958	935	852
propanal-D	Aldehyde	2042	2099	2118	2007	1901	1734
Acetone	Ketones	2935	2954	2943	2971	2949	3238
E-2-Pentenal-M	Aldehyde	930	929	920	376	361	331
E-2-Pentenal-D	Aldehyde	109	141	145	179	161	200
Nonanal	Aldehyde	61	62	67	56	59	61
E-2-heptenal	Aldehyde	28	69	73	119	124	160
E-2-hexenal	Aldehyde	64	78	71	92	86	99
1-Propanol-M	Alcohols	425	412	399	26	25	14
1-Propanol-D	Alcohols	87	82	86	97	84	127
Tetrahydrofuran	Heterocycles	516	518	511	1019	859	1262
Ethane-1-1-diethoxy-	Aldehyde	411	397	366	415	347	201
ButyrAldehyde-M	Aldehyde	450	454	439	468	336	355
ButyrAldehyde-D	Aldehyde	232	255	253	267	250	157
Ethyl Acetate-M	Esters	747	706	681	634	446	470
Ethyl acetate-D	Esters	3088	3109	3077	3060	3045	3037
2-Propanol-M	Alcohols	482	418	401	234	144	288
2-Propanol-D	Alcohols	181	162	154	120	115	312
isobutyl acetate	Esters	188	208	174	80	74	94
Methyl 2-methylbutanoate	Esters	98	100	93	150	133	164
Ethyl butyrate-M	Esters	93	99	86	236	222	300
Ethyl butanoate-D	Esters	11	14	9	247	219	285
2-Butanol-M	Alcohols	142	131	122	12	56	77
2-butanol-D	Alcohols	17	15	20	116	119	143
1-Propanol-2-methyl	Alcohols	14	13	12	104	97	134
2-methyl-2-pentenal	Aldehyde	13	17	19	163	155	187
n-heptanal-M	Aldehyde	1061	1319	1381	1883	1844	1881
heptanal-D	Aldehyde	8	12	14	76	68	102
E-2-pentenal	Aldehyde	164	182	171	785	784	842
butyl butanoate	Esters	131	129	121	57	53	55
acetic acid-D	Acids	35	32	26	22	26	29
Pentanol	Alcohols	113	131	140	128	108	120
2-Butanone-M	Ketones	471	464	459	489	206	141
2-Butanone-D	Ketones	245	269	277	350	329	310
52		1113	1148	1152	1116	1096	311
53		276	141	129	163	179	692
54		60	60	63	300	292	283
55		30	22	30	228	203	181
56		21	18	19	102	84	111
57		91	109	99	96	92	19
58		35	36	32	135	137	151
59		83	49	50	55	41	219
60		31	24	18	26	28	139
61		97	96	98	230	170	241
62		48	62	70	74	212	255
63		28	40	49	32	154	187
64		74	41	47	52	49	181
65		91	103	106	213	194	175
66		12	34	36	38	190	208
67		17	17	13	19	182	176
Total		29400	30528	30379	31949	31112	31370

[a] F1: Control group,F2: Experimental group.
